# Oral administration of *Lactobacillus paracasei* NCC 2461 for the modulation of grass pollen allergic rhinitis: a randomized, placebo-controlled study during the pollen season

**DOI:** 10.1186/s13601-015-0085-4

**Published:** 2015-12-09

**Authors:** Chiara Nembrini, Anurag Singh, Carlos Antonio De Castro, Annick Mercenier, Sophie Nutten

**Affiliations:** Nestlé Research Center, Nestec Ltd, PO Box 44, 1000 Lausanne-26, Switzerland

**Keywords:** Probiotic, Allergy, Symptoms, Nasal, Allergen, Exposure

## Abstract

**Background:**

The efficacy of *Lactobacillus paracasei* NCC 2461 in modulating allergic rhinitis was previously evaluated in two exploratory clinical studies. Oral administration with NCC 2461 reduced specific subjective symptoms following nasal provocation tests with controlled grass pollen allergen concentrations. Our aim was to confirm the anti-allergic effect of NCC 2461 in grass pollen allergic subjects exposed to natural doses of allergens during the pollen season.

**Findings:**

A double-blind, randomized, placebo-controlled, parallel study was conducted with 131 grass pollen allergic subjects from May to July 2012 in concomitance with the pollen season in Berlin. NCC 2461 or placebo was administered daily for an 8-week period to adult subjects with clinical history of allergic rhinitis to grass pollen, positive skin prick test and IgE to grass pollen. During the 8 weeks, symptoms and quality of life questionnaires were filled out, and plasma was collected for IgE analysis at screening and at the end of the intervention. All subjects were included within a 5-day interval, ensuring exposure to similar air pollen counts for each individual during the trial period. The results obtained show that symptoms increased with pollen loads, confirming a natural exposure to the allergen and presence of pollen-induced allergic rhinitis in the subjects. However, no significant differences were observed in allergic rhinitis symptoms scores, quality of life, or specific IgE levels between subjects receiving NCC 2461 as compared to placebo administration.

**Conclusion:**

In contrast to previous findings, oral administration of NCC 2461 did not show a beneficial effect on allergic rhinitis in a field trial. The influence of study design, allergen exposure and intervention window on the efficacy of NCC 2461 in modulating respiratory allergy should be further evaluated.

**Electronic supplementary material:**

The online version of this article (doi:10.1186/s13601-015-0085-4) contains supplementary material, which is available to authorized users.

## Findings

### Probiotics and allergic rhinitis

 Allergic rhinitis to grass pollen affects around 20 % of the population in Europe, USA and Oceania and is characterized by nasal and ocular symptoms, such as nasal congestion or watery eyes, which significantly impact the quality of life of allergic subjects [1–3]. In addition to standard medications, such as anti-histamines and corticosteroids, aiming at the management of allergic rhinitis symptoms, nutritional interventions with probiotics have been explored in clinical trials [4, 5]. Oral intake of probiotics, in particular lactobacilli and bifidobacteria, has been shown to modulate allergic immune responses as well as to have a significant impact on symptoms reduction [6–11]. Administration of *Lactobacillus paracasei* NCC 2461 significantly decreased subjective nasal congestions in subjects with a medical history of allergic rhinitis to grass pollen, as evaluated following a nasal provocation test (NPT) in a proof-of-concept study performed outside of the pollen season [12]. In a second study, NCC 2461 improved NPT-induced nasal pruritus in grass pollen allergic subjects when compared to a different probiotic blend; the effect observed was comparable to the symptoms reduction obtained with an anti-allergic drug tested with the same NPT protocol [13]. In line with these results, we aimed to further demonstrate the efficacy of NCC 2461 in a clinical trial performed during the grass pollen season, in which subjects are exposed to natural doses of allergens.

### Study design and outcomes

The study was designed as a randomized, double blind, placebo-controlled, parallel trial, and was performed between May and July 2012 at Charité Research Organisation GmBh in Berlin (ClinicalTrials.gov: NCT01653652). One hundred and thirty-one volunteers were enrolled on the basis of the following inclusion criteria: 18–65 years of age, body mass index in the range of 19–32 kg/m^2^, established allergic rhinitis to grass pollen for more than 2 years, positive skin prick test to grass pollen and grass pollen-specific IgE (≥0.35 kU/L). Exclusion criteria comprised pregnancy, infection or antibiotic intake at time of enrollment, asthma and other chronic disorders, and the use of systemic corticosteroids prior to randomization. Enrollment was completed within 5 days to ensure that all subjects were exposed to natural airborne pollen during the same period of the pollen season. The treatment consisted of daily oral intake of 5 g of placebo (maltodextrin) or a probiotic blend containing 5 × 10^9^ CFU NCC 2461 over a period of 8 weeks. Placebo and probiotics were packed in powder form in single sachets for each daily dose. Study design and CONSORT flow diagram are outlined in Additional file 1: Figure S1.

 The primary objective of the study was to evaluate the efficacy of NCC 2461 in mitigating grass pollen-induced allergic rhinitis symptoms by comparing daily total nasal symptom score (TNSS) over 8 weeks (from V1–V3) between the probiotic and the placebo group. TNSS as well as total ocular symptom score (TOSS, both described in Additional file 1: Questionnaires and plasma analysis) were recorded daily by the subjects with the help of an electronic patient reported outcome (ePRO) device. On a weekly basis, subjects were asked to fill a validated quality of life questionnaire specific for allergic rhinitis, the mini Rhinoconjuctivitis Quality of Life Questionnaire (miniRQLQ) [14]. Subjects were allowed to take any anti-allergic medication at their discretion, and the type and dose of each drug was recorded. Decrease in medication intake through probiotic intervention was a secondary objective of the study, and the outcome measure was the weekly rescue medication score questionnaire (Additional file 1: Questionnaires and plasma analysis). Of note, TNSS was adjusted for medication score at V1, to avoid possible biases on symptoms of the basal status of the subjects.

To evaluate the effect of probiotic administration on grass pollen-specific IgE, serum was collected at screening and at V3 and results compared between NCC 2461 and placebo group.

The primary statistical analysis was carried out on the change from baseline TNSS scores using a Linear Mixed Model, where treatment group, day of treatment and their interaction were considered as fixed effects and with subject number as a random effect to control for within subject variability. Secondary analyses were done in a similar fashion as the primary analysis. A summary of inclusion and exclusion criteria, primary and secondary outcomes, as well as methods can be found in the Additional file 1.

### Efficacy of *L. paracasei* NCC 2461

Baseline demographics are shown in Additional file 1: Table S1. Sixty-eight subjects were assigned to the placebo group, sixty-three to the NCC 2461 group, and all one hundred and thirty-one subjects were included in the intention-to-treat (ITT) analysis. Of note, results of the per-protocol analysis did not differ from ITT. One subject receiving placebo withdrew from the study due to a bacterial infection unrelated to product intake.

**Fig. 1:**
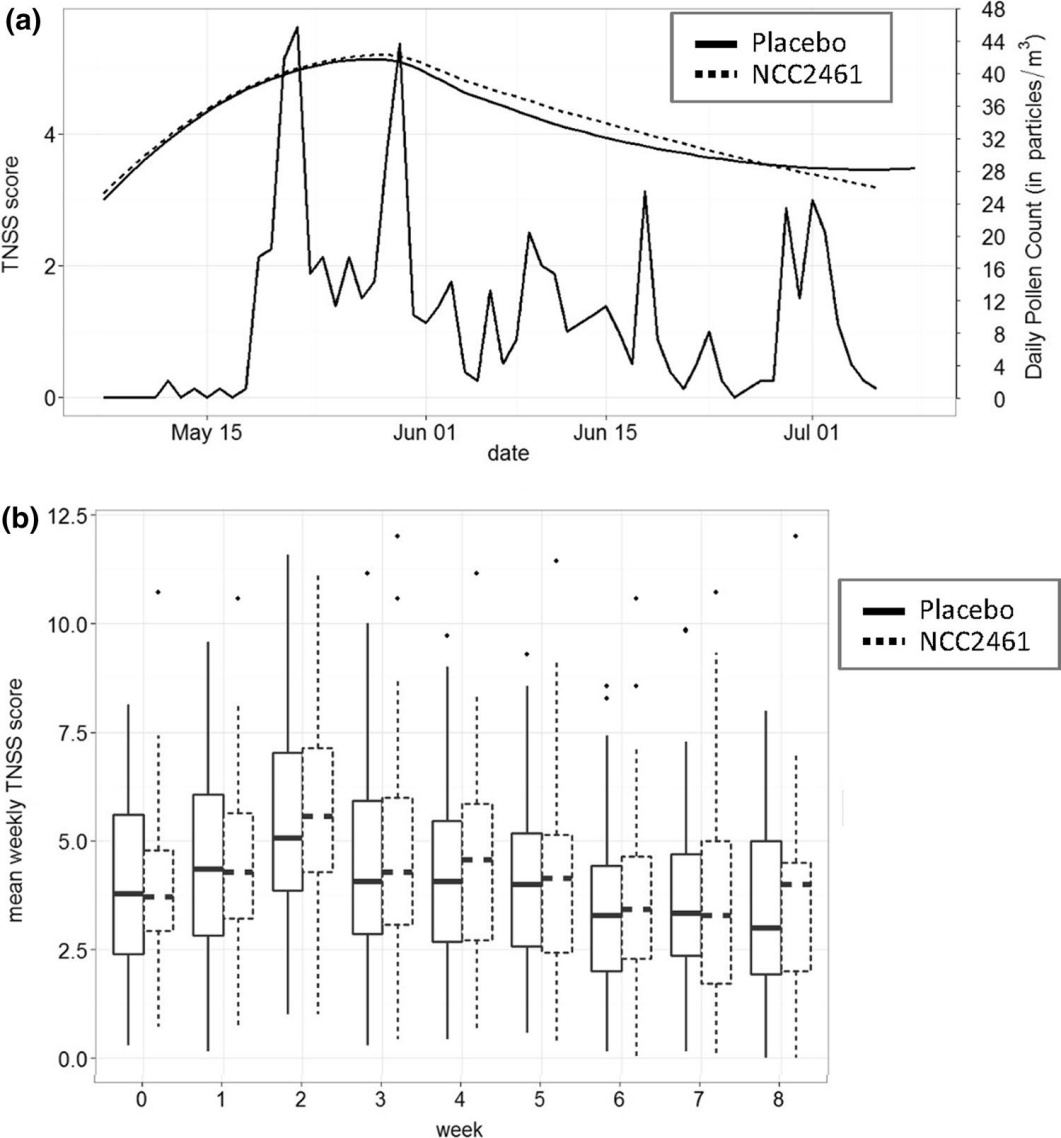
TNSS is comparable between placebo and NCC 2461 administration. **a** The correlation between daily grass pollen counts during the trial period and daily TNSS values is represented; both parameters reached maximum values in the last week of May, 20 days after the start of the trial, and no significant differences were observed between the study groups. The *solid* and *broken curves* represent Loess local smoothing method (with 95 % level of confidence) applied to TNSS scores of placebo and NCC 2461 groups, respectively, in order to summarize daily symptom scores. The *volatile line* refers to daily pollen counts with corresponding levels (referring to the *right vertical axis*). Pollen data were provided by the Geowissenschaften Institut für Meteorologie of the Freie Universität in Berlin. **b** Mean TNSS score for each week of each subject was calculated; *boxplots* represent the weekly mean for the two study groups

**Fig. 2:**
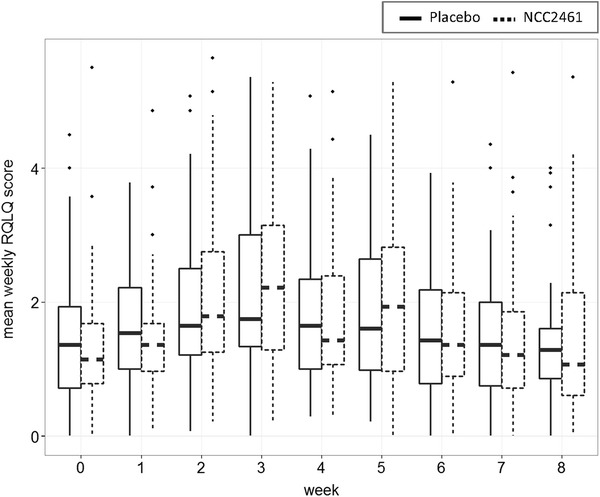
NCC 2461 administration does not impact quality of life. Similar to TNSS, no statistically significant differences were observed between probiotic and placebo group at any time point. *Boxplots* for weekly mean miniRQLQ are shown

Analysis of grass pollen concentrations in Berlin during the 2012 season indicated that at enrollment, beginning of May, pollen counts were still low (Fig. 1a). Further analysis highlighted a positive correlation between pollen counts and the primary outcome TNSS confirming the presence of grass pollen-induced allergic rhinitis manifestations in line with the clinical history of the study subjects. The maximum values for both pollen and symptom measures were observed 20 days after the start of the trial. Treatment with NCC 2461 however did not have an effect on symptoms, as no significant differences in TNSS were observed between probiotic and placebo at any time point (Fig. 1b). Individual symptoms scores were also comparable between groups (Additional file 1: Figure S2). In line with these results, NCC 2461 administration did not show an effect on quality of life over the 8-week period (Fig. 2), and grass pollen-specific IgE in plasma did not differ between placebo and probiotic group at both time points assessed (Additional file 1: Figure S3). Total and individual ocular symptoms were also comparable (data not shown). Subjects were allowed to take anti-allergic medication whenever they felt the need; the intake was not influenced by the probiotics, as no statistically significant differences in frequencies were observed when compared to placebo at any of the time points (Table 1).

**Table 1:** Medication score comparison between NCC 2461 and placebo group

Placebo									
Week	0 (N = 68)	1 (N = 68)	2 (N = 68)	3 (N = 68)	4 (N = 68)	5 (N = 68)	6 (N = 67)	7 (N = 67)	8 (N = 41)
Medication score									
0	63 % (43)	68 % (46)	59 % (40)	54 % (37)	63 % (43)	65 % (44)	63 % (42)	72 % (48)	66 % (27)
1	32 % (22)	29 % (20)	37 % (25)	38 % (26)	34 % (23)	31 % (21)	31 % (21)	25 % (17)	29 % (12)
2	1 % (1)	1 % (1)	1 % (1)	3 % (2)	1 % (1)	3 % (2)	4 % (3)	1 % (1)	2 % (1)
3	3 % (2)	1 % (1)	3 % (2)	4 % (3)	1 % (1)	1 % (1)	1 % (1)	1 % (1)	2 % (1)

NCC 2461									
Week	0 (N = 63)	1 (N = 63)	2 (N = 63)	3 (N = 63)	4 (N = 63)	5 (N = 63)	6 (N = 63)	7 (N = 63)	8 (N = 35)
Medication score									
0	81 % (51)	84 % (53)	63 % (40)	63 % (40)	65 % (41)	63 % (40)	70 % (44)	70 % (44)	74 % (26)
1	19 % (12)	16 % (10)	35 % (22)	32 % (20)	33 % (21)	35 % (22)	29 % (18)	29 % (18)	26 % (9)
2	0 % (0)	0 % (0)	0 % (0)	3 % (2)	0 % (0)	0 % (0)	0 % (0)	0 % (0)	0 % (0)
3	0 % (0)	0 % (0)	2 % (1)	2 % (1)	2 % (1)	2 % (1)	2 % (1)	2 % (1)	0 % (0)

Numbers in brackets are frequencies

Probiotic administration was considered safe as no serious adverse event was recorded during the trial. Subjects receiving NCC 2461 reported fewer episodes of nasopharyngitis, however the frequency of minor adverse events was not a trial outcome and was therefore not considered in the statistical analysis.

### Discussion

Prior to this study, the effect of NCC 2461 in modulating grass pollen allergic rhinitis was evaluated in two exploratory studies, outside the pollen season in nasal provocation test settings [12, 13]. Results of these trials suggested a role of NCC 2461 in immune regulation, as cytokine expression by peripheral blood mononuclear cells cultures [12] or in nasal secretions [13] was significantly different when compared to a placebo [12] or a different probiotic blend [13]. In addition, both studies showed that NCC 2461 administration might affect distinct subjective symptoms of allergic rhinitis, namely congestion [12] and pruritus [13]. In contrast, the results from the study presented in this manuscript indicate no effect of NCC 2461 in modulating TNSS, TOSS, nor any of the symptoms considered individually. The discrepancy in outcomes between the different studies using NCC 2461 might be explained by the difference in study design between the previous trials and the current one, and the different approaches taken to expose subjects to the allergen in order to induce allergic rhinitis symptoms.

Wassenberg et al. [12] as well as Perrin et al. [13] evaluated the effect of NCC 2461 administration on symptoms’ reduction following a NPT in which all subjects were exposed to the same increasing doses of grass pollen at each challenge; both trials were performed outside of the pollen season. In our current in-season trial, exposure of each subject to grass pollen occurred in a natural and obviously non-controlled manner, and allergen doses might have varied considerably over time as well as within the whole study population. In addition to pollen counts, the country in which the study was performed, as well as other environmental factors such as climate and air pollution could also influence the results of a trial conducted during the pollen season [15, 16].

The difference in outcome measures between studies should also be considered; in the previous trials subjective symptoms (pruritus and nasal congestion) were measured with visual analogue scales, and immunological parameters such as cytokine secretion and IgG4 were not measured in the study performed in Berlin. Also, the dose we used here (5 × 10^9^ CFU/day) was slightly lower than in previous NCC 2461 studies due to the different format used; however, it was still in the range of common doses of probiotics tested in clinical trials [6, 9, 17, 18].

Another important variable to take into account is the use of anti-allergic medication by the subjects. Intake was recorded and compared between probiotic and placebo group through the rescue medication score questionnaire; no significant differences could be observed. On the other hand, there was no adjustment of the primary endpoint TNSS for covariates such as anti-allergic medication intake at any time point except for V1, to correct for basal level. For this reason we cannot exclude that the single consumption of anti-histamines or corticosteroids by the subjects had an influence on the reporting of their symptoms. In this case, a primary outcome comprising both symptoms and medication intake score results could have been a relevant approach [19]. Moreover, allergic rhinitis to other aeroallergens such as house dust mite or pet dander was not an exclusion criterion, as the majority of subjects suffering from allergic airway diseases are polysensitized; thus, reactions other than to grass pollen could have had an impact on TNSS and medication score as well.

Our objective was to evaluate the role of NCC 2461 in conditions as near as possible to real-life, namely with natural exposure to allergens and with medication intake at subject’s discretion. It can be speculated however that the effect of the intervention is more difficult to detect in these settings. Of note, in three clinical trials in which probiotic administration was associated to the controlled intake of anti-allergic drugs such as loratadine or levocetirizine, a significant impact on symptoms reduction or quality of life improvement on top of the medication was observed [6, 18, 20]. The trials described in Costa et al. and Lue et al. were performed in subjects with moderate-to-severe rhinitis according to ARIA classification; significant clinical differences might be better detectable in this population as compared to mild allergic rhinitis [21, 22]. In our trial, we did not differentiate subjects on the basis of ARIA classification.

Importantly, in contrast to our earlier NCC 2461 trials, in which probiotics were administered for 4 weeks prior to pollen challenge by NPT [12, 13], probiotic intake in the study presented here occurred at the same time as the exposure to the allergen. In fact in most of previously published studies in which probiotics had a positive impact on allergic rhinitis to pollen (grass, birch or Japanese cedar), the intervention started weeks before season start or nasal challenge [11–13, 17, 22–25]. On the other hand, in the only other reported trial that had a similar experimental design as the current one, i.e., parallel, 8-week long, with similar outcomes and most importantly run at the peak of the pollen season, we could show that administration of *Bifidobacterium lactis* NCC 2818 did significantly reduce TNSS in allergic subjects [9]. The mechanisms of action of probiotics, including NCC 2461, are not entirely elucidated and can vary according to the strain used; the same is true for the appropriate doses and intervention windows needed to observe a beneficial effect [21, 22, 26–31]. It can be speculated that some probiotics have a higher impact on allergic responses by priming the immune system prior to allergen challenge; this could be the case of NCC 2461. Different strains might conversely be more effective in symptoms management when the allergy reaction is already ongoing. The immune-regulatory properties and mechanisms of action of specific probiotic strains are thus worth being carefully investigated; study design and intervention window of future efficacy trials should also be aligned accordingly.

Finally, we believe more evidence is needed in closer-to-real-life, field-trials settings to conclude on the most appropriate probiotic intervention. Taken together however, our NCC 2461 trials and other previously published observations suggest that starting probiotic intake before the pollen season as well as administration together with a controlled medication regimen may be the preferable strategy for the management of seasonal allergic manifestations.

## Additional file

10.1186/s13601-015-0085-4 Population, protocols and supplementary figures.

## Abbreviations

ARIA: allergic rhinitis impact on asthma
CFU: colony forming unit
ePRO: electronic patient reported outcome
ITT: intent-to-treat
NCC: Nestlé culture collection
NPT: nasal provocation test
RQLQ: rhinoconjunctivitis quality of life questionnaire
TNSS: total nasal symptom score
TOSS: total ocular symptom score
