# Supplementary material for: Oral administration of Lactobacillus paracasei NCC 2461 for the modulation of grass pollen allergic rhinitis: a randomized, placebo-controlled study during the pollen season
Source: Clin Transl Allergy. 2015 Dec 9;5:41. doi: 10.1186/s13601-015-0085-4 (PMC4673783; doi:10.1186/s13601-015-0085-4)
Supplement: Supplementary file 1 — 10.1186/s13601-015-0085-4 Population, protocols and supplementary figures. [file 13601_2015_85_MOESM1_ESM.docx]

**Additional File 1**

**Table S1. Baseline demographics.** Numbers after percents are frequencies. *a, b, c* represent the lower quartile *a*, the median *b*, and the upper quartile *c* for continuous variables.

|  | **Placebo**  **N = 68** | **NCC2461**  **N = 63** |
| --- | --- | --- |
| Sex  Male  Female | 50% (34)  50% (34) | 48% (30)  52% (33) |
| Weight strata  (45,54]  (54,64]  (64,74]  (74,103] | 9% (6)  25% (17)  29% (20)  37% (25) | 10% (6)  29% (18)  21% (13)  41% (26) |
| Age strata  (18,30]  (30,50]  (50,65] | 53% (36)  40% (27)  7% (5) | 59% (37)  35% (22)  6% (4) |
| Grain pollen allergy  No  Yes | 0% (0)  100% (68) | 0% (0)  100% (63) |
| Seasonal allergy  No  Yes | 0% (0)  100% (68) | 0% (0)  100% (63) |
| Age (years) | 25.0^a^ 30.0^b^ 42.0^c^ | 25.0 29.0 43.5 |
| Height (cm) | 168.00 176.00 182.25 | 166.00 173.00 180.00 |
| Weight (kg) | 60.0 69.5 82.0 | 60.0 69.0 79.5 |
| Systolic blood  pressure (mmHg) | 112.75 122.00 133.25 | 112.50 120.00 130.50 |
| Diastolic blood  pressure (mmHg) | 62.00 68.00 76.25 | 65.00 71.00 77.50 |
| Heart rate (bpm) | 69.00 77.50 86.25 | 68.50 75.00 82.00 |

**Figure S1. Study flow chart.** (a) Study started beginning of May 2012 with V1 and continued for 8 weeks until V3. At V2, compliance was checked and product for the following 4 weeks distributed. Blood was drawn at V0 and V3. Arrows represent daily product intake and recording of TNSS and TOSS (solid) and weekly recording of miniRQLQ and medication score (dashed). (b) CONSORT flow diagram; 63 subjects were assigned to probiotic arm, 68 to placebo arm. One subject in the placebo group withdrew from the study.

**
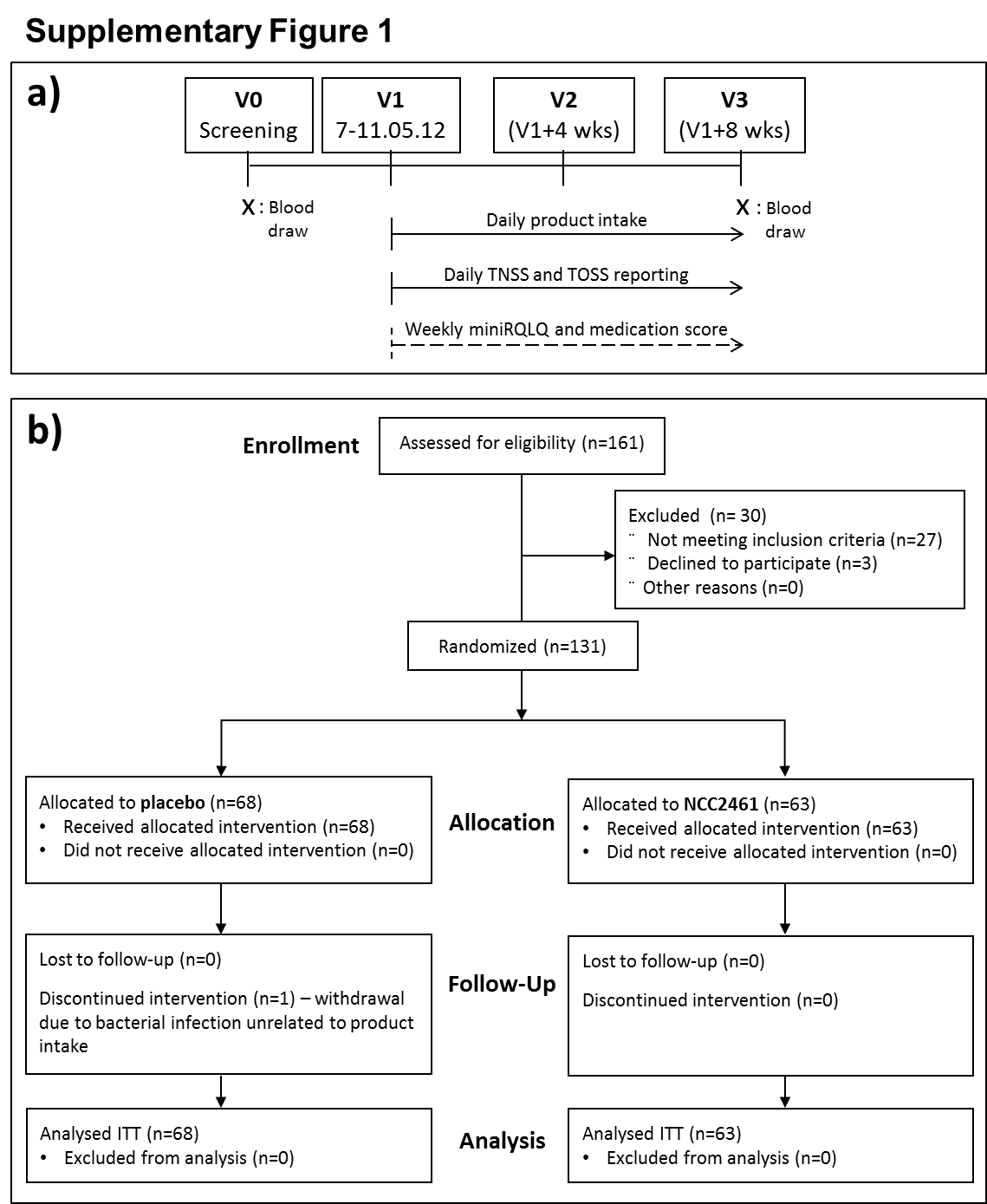
**

**Figure S2. Individual single scores are comparable between placebo (solid line) and probiotic (broken line).** Means for each group across the different visits and segmented according to the 4 components of the TNSS score are represented. Differences in means are at most around 0.1 in magnitude.


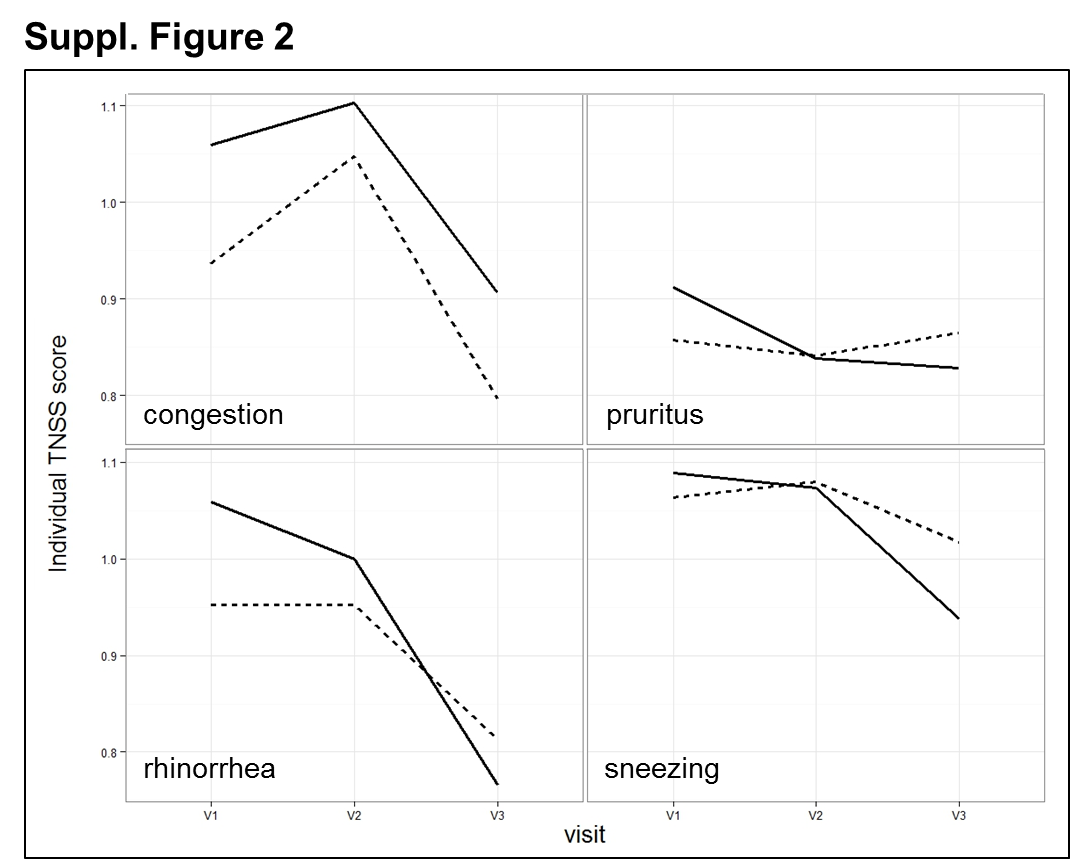


**Figure S3. NCC2461 administration does not impact grass-pollen specific IgE.** Grass pollen-specific IgE was measured in the plasma at V0 and V3 by ImmunoCAP. Values were comparable at both visits between NCC2461 and placebo group. Boxplots for the natural logarithm of IgE concentrations by visit are shown.


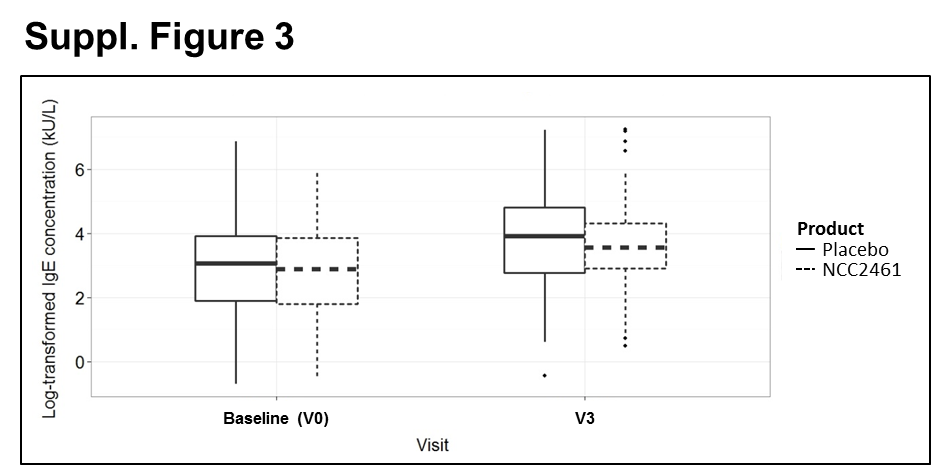


**Population and study protocol**

One hundred and thirty-one subjects were invited to participate in the study (Registration number: NCT01653652) and signed written inform consent. Inclusion criteria were: a) 18 to 65 years of age at the time of enrolment, b) Body Mass Index in the range 19-32 kg/m^2^, c) established seasonal allergic rhinitis (clinical history of rhinorrhea, congestion, sneezing, pruritus for more than 2 years), d) positive skin prick test (wheal diameter in response to grass pollen > 2mm), e) presence of specific IgE to grass pollen (≥ 0.35 kU/L), f) signature of consent form, g) agreement to not take any probiotic containing products except the study product for the whole duration of the study. Exclusion criteria comprised: a) current treatment with antibiotics or undergoing allergen-immunotherapy, b) pregnancy, c) vasomotor rhinitis, nasal cavity disorders (nasal polyps), ear infections (otitis media), d) other chronic diseases (e.g. gastrointestinal, cardiovascular, infections), e) asthma, f) participation in another interventional clinical trial (current or for the preceding 2 months), g) chronic use of systemic corticosteroids prior to randomization. Subjects were either assigned to NCC2461 group (63 subjects) or placebo (68 subjects). The protocol was approved by the Ethics Commission of the Charité University Hospital in Berlin, Germany (Ethikkommission der Charité – Universitätsmedizin, Berlin) and all enrolled subjects provided informed consent before the start of the study.

**Study product**

The treatment consisted of a daily dose of 5x10^9^ CFU *Lactobacillus paracasei* NCC2461 (CNCM I-2116, ST11, Nestlé, Switzerland) blended in maltodextrin, or maltodextrin only (placebo). Study products were packed in powder form in 5 g sachets for daily intake, and were similar in appearance and color; subjects were asked to mix the content of the sachet with a drink such as milk, water or juice. Both probiotic and placebo were produced at PTC Konolfingen (Nestec, Switzerland). At V1 and V2, each subject received the equivalent number of 5 g sachets needed for the following 4 weeks of trial. Compliance with respect to product intake was checked at V2 and V3 by counting empty and remaining sachets.

**Questionnaires and plasma analysis**

The primary objective of the trial was to assess the efficacy of oral administration of NCC2461 on mitigation of seasonal allergic rhinitis symptoms. Primary endpoint was TNSS comparison over 8 weeks (from V1 to V3) between probiotic treatment and placebo. TNSS was recorded daily on a scale of 0 to 12 as a sum of the following four individual symptoms: congestion, rhinorrhea (runny nose), pruritus (itching) and sneezing. Each symptom had to be scored on a scale of 0 to 3, whereby 0 = no symptom, 1 = mild symptom (present but easily tolerated), 2 = moderate symptom (bothersome but tolerated) and 3 = severe symptom (hard to tolerate).

Secondary objectives included the assessment of NCC2461 safety and the effect of probiotic administration on improving the following parameters from V1 to each follow-up visit (V2 and V3): quality of life, total ocular symptoms, total symptoms (i.e. the sum of nasal and ocular symptoms), each individual nasal and ocular symptoms, and frequency of anti-allergic medication intake. Secondary endpoints for these objectives, measured between V1 and V3, were miniRQLQ (1), TOSS and medication score questionnaire. TOSS was recorded daily on a scale of 0 to 9 as a sum of the following individual symptoms: redness, watery eyes, and itching; each symptom had to be scored on a scale of 0 to 3, as in TNSS (see above). To calculate the medication score on a scale of 0 to 3, subjects had to indicate the intake of anti-allergic medication for the preceding week, whereby 0 = none, 1 = use of oral anti-histaminics and nasal drops (excluding corticosteroids), 2 = use of corticosteroids (oral or local), 3 = use of oral anti-histaminics and steroids.

All questionnaires and scores were recorded by the subjects with the help of a provided electronic Patient Reported Outcome (ePRO) device (CRF Health, USA); site personnel and subjects were properly trained to use the ePRO device at study start.

Specific IgE to grass pollen was measured at V0, for evaluation of inclusion criteria, and at V3. Values were compared between NCC2461 and placebo group at both time points as secondary outcome. Grass pollen-specific IgE were measured after plasma collection with ImmunoCAP (Thermo Fischer Scientific, Germany).

**Statistical analysis**

Sample size calculation for this trial was based on previous results obtained with the primary outcome (2). With an estimated decrease of 20% in TNSS in the probiotic groups as compared to the control, a sample size of 66 subjects per arm, including 15% drop-out rate, was judged sufficient to detect statistically significant differences. The primary statistical analysis was carried out on the change from baseline TNSS scores using a Linear Mixed Model, where treatment group, day of treatment and their interaction were considered as fixed effects and with subject number as a random effect to control for within subject variability; the time was considered as a continuous variable since the questionnaire were filled daily. Primary Endpoint was adjusted for medication score at V1. Statistical analysis for RQLQ scores were done in a similar fashion as the primary analysis, taking the time as categorical aggregating individual weekly scores. The statistical analysis on change from baseline of IgE concentration was done also with a Linear Mixed model having applied logarithmic transformation. Statistical analysis was performed using R, version 2.15.1, and © SAS software, version 9.3 of the SAS System for Windows (SAS Institute Inc., USA).

**References**

1. Juniper, E. F., Thompson, A. K., Ferrie, P. J., & Roberts, J. N. (2000) *Clin. Exp. Allergy.* **30**, 132-140.

2. Singh, A., Hacini-Rachinel, F., Gosoniu, M. L., Bourdeau, T., Holvoet, S., Doucet-Ladeveze, R., Beaumont, M., Mercenier, A., & Nutten, S. (2013) *Eur. J. Clin. Nutr.* **67**, 161-167.
